# Follow-Up of Robotic Mitral Valve Repair: A Single Tertiary Institution Experience in China

**DOI:** 10.1155/2022/1997371

**Published:** 2022-05-21

**Authors:** Guang Li, Ping Li, Shuo Liu, Bin You

**Affiliations:** Department of Cardiac Surgery, Beijing Anzhen Hospital, Capital Medical University; Beijing Institute of Heart, Lung and Blood Vessel Diseases, Beijing 100029, China

## Abstract

Surgical treatment of mitral valve diseases has become minimally invasive. This study analyzed the follow-up results of patients after mitral valve repairs (MVRep) using the da Vinci robot. The clinical data of patients who underwent minimally invasive MVRep using the da Vinci robot between January 2016 and June 2021 and completed follow-ups were prospectively collected. All operations were performed by the same surgeon and assistants. The data of a total of 120 patients were available for analysis, including 78 males (65%) and 42 females aged 49.9 ± 12.1 years (range, 19–73 years). Among them, there were 30 cases (25%) of mitral valve prolapse, 87 cases (72.5%) of mitral regurgitation, and 40 cases of combined tricuspid regurgitation. Edwards Physio II annuloplasty rings were implanted intraoperatively, followed by continuous sutures. The intraoperative cardiopulmonary bypass time was 152.32 ± 45.77 min, and the aortic occlusion time was 95.13 ± 5.64 min. After surgery, patients were followed up regularly with echocardiography with a follow-up period of 3–57 months postoperatively. One patient died in the early stage, and five patients required sternotomy due to postoperative bleeding. Follow-up transesophageal echocardiography showed that the end-systolic diameter, end-diastolic diameter, and ejection fraction of the left ventricular all improved after surgery. Among Chinese patients, MVRep using the da Vinci robot is a safe and effective surgical approach.

## 1. Introduction

In 2007, Gao et al. completed the first da Vinci robot-assisted mitral valve repair (MVRep) in China, marking the beginning of the era of domestic robotically assisted heart valve surgeries, which has continued now for 15 years [1, 2]. Robotic cardiac surgery both enables more precise surgical positioning and increases surgical accuracy. Compared to traditional MVRep with median sternotomy, robotic cardiac surgery minimizes the surgical incision and reduces the incidence of surgically related trauma and infection [3]. Some studies indicated that robotic MVRep was equivalent to traditional sternotomy in terms of mortality, comorbidities, and long-term durability [4–6]. A relatively large number of patients have received robotic-assisted MVRep and postoperative follow-up in our hospital in China. This study aimed to analyze the patients' follow-up results after robotic MVRep.

## 2. Patients and Methods

A total of 121 patients who underwent da Vinci robot-assisted MVRep between January 2016 and June 2021 were selected. All adult patients suffering from MV disorders were considered to be candidates for robotic repair. Patients with severe pulmonary hypertension, severe left ventricular dysfunction (ejection fraction less than 20%), or coronary artery disease necessitating multiple vessel bypass grafting were excluded from the study. One patient was excluded for being <18 years of age. A total of 120 adult patients were included in the da Vinci group. Ethical committee approval was obtained from Beijing Anzhen Hospital, and written informed consents were obtained from all patients.

### 2.1. Surgical Procedures

The mitral valve conditions were routinely assessed preoperatively by transthoracic echocardiography (TTE) [7]. All operations were performed by the same surgeon and assistants. After successful general anesthesia, the patient was placed in the supine position with the right side elevated by 30°. Routine disinfection was performed and sterile towels were placed. The right femoral artery and vein as well as the right internal jugular vein were cannulated to establish closed extracorporeal circulation. An incision was made along the right anterior axillary line through the fourth intercostal space on the skin, subcutaneous tissue, and muscle layer. The anesthesiologist was instructed to access the chest after the right lung collapsed. The pericardium was longitudinally incised 2 cm superior to the left phrenic nerve and suspended. A hole was made along the anterior axillary line at the third intercostal space to retract the suture on the right pericardium out of the body. Meanwhile, a carbon dioxide tube was cannulated through the hole at the third intercostal space to insufflate carbon dioxide gas intraoperatively. A purse-string suture was performed through the ascending aorta and left superior pulmonary vein. After heparinization at 3 mg/kg, a perfusion tube and a drainage tube were inserted into the left heart. Holes were made in the second and fourth intercostal spaces at the right midclavicular line as well as in the sixth intercostal space at the right anterior iliac line [8–11].

A da Vinci robotic arm was placed to establish the robotic surgery system. The machine was turned on, and the patient was cooled. A Chitwood occlusion clamp was inserted through the auxiliary hole in the third intercostal space to occlude the ascending aorta. Cardioplegia was perfused, and ice saline was perfused into the pericardium in the meantime for cooling. After cardiac arrest occurred, the left atrium was incised through the interatrial groove. The mechanical arm of the da Vinci robot was placed on the retractor of the left atrium to expose the mitral valve. A mitral valve annuloplasty ring (Edwards Physio II) was placed. After the leakage test results improved, the interatrial groove incision was closed.

**Figure 1:**
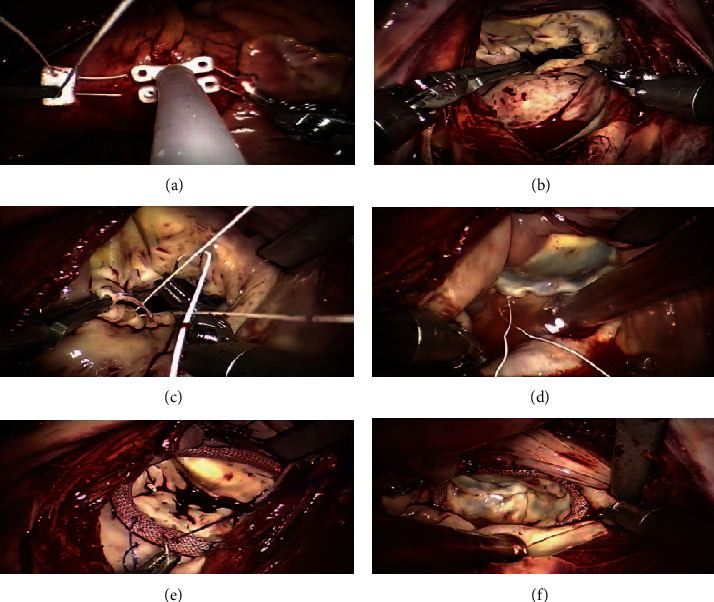
Key surgical operation steps of robotic mitral valve repair. (a) After the Chitwood clamp was placed, insert the perfusion catheter; (b) Incise the left atrium along the interatrial groove to explore the mitral valve; (c) Form the artificial chordae; (d) Perform a leakage pressure test in the left ventricle; (e) Place the Edwards Physio II annuloplasty ring, followed by a continuous suture; (f) Perform a leakage pressure test in the left ventricle after annuloplasty.

Postoperatively, the patient was rewarmed, and the ascending aorta was opened. The heart started beating again, and parallel circulation was established while the machine was gradually turned off. The cannulae were removed, hemostasis was achieved, and the pericardium was closed. After intraoperative transesophageal echocardiography (TEE) results were confirmed as satisfactory, the heparin was neutralized with protamine and the chest was closed layer by layer. TEE may be performed postoperatively to re-evaluate the valvuloplasty outcome. A TEE result suggesting a small amount or less of mitral regurgitation (MR) indicated a successful mitral valvuloplasty.  Figure 1 demonstrates the key surgical steps.

### 2.2. Postoperative Treatment and Follow-Ups

Systemic therapies such as analgesia, anticoagulation, cardiotonic agents, and diuresis were provided postoperatively. The chest tube was removed after the drainage volume met the indications for extubation. The patient was discharged once the vital signs returned to normal. After discharge, patients were regularly followed up via telephone. During the follow-up period, complications were recorded.

### 2.3. Statistical Analysis

All statistical analyses were performed by SPSS (V.19). Continuous data is reported as the mean standard deviation, whereas categorical data is provided as proportions.

## 3. Results

A total of 120 patients were included in the study, of whom 30 (25%) had mitral valve prolapse, 87 (72.5%) had MR, and 40 (33.3%) had combined tricuspid regurgitation. The intraoperative cardiopulmonary bypass time was 152.32 ± 45.77 min, and the aortic occlusion time was 95.13 ± 45.64 min. The operative duration was 5.34 ± 1.31 h (Table 1). There were no cases of intraoperative conversion to sternotomy for valve repair.

### 3.1. Follow-Up TEE

Follow-up TEE showed that the left ventricular end-systolic diameter was 32.0 ± 4.91 mm, the end-diastolic diameter was 47.8 ± 5.6 mm, and the ejection fraction was 59.5 ± 7.0%, which were significantly improved compared with preoperative levels.

### 3.2. Postoperative Complications

Early reoperation was defined as reoperation within 30 days after surgery. The most common reoperation was sternotomy for the re-exploration for bleeding in 5 cases (4.2%). One patient died of a pleural effusion in the early postoperative period.

### 3.3. Follow-Up

Patients were followed up for 3–57 months postoperatively. Hypertrophic obstructive cardiomyopathy was observed in 6 patients (5%), while Barlow syndrome was observed in 8 (6.7%).

## 4. Discussion

Robotic surgery has been a major focus for cardiac surgeons. Robotic MVRep has been widely implemented in Europe and the United States as well as in Asian countries [12–17]. This study reported the clinical data and follow-up results of 120 Chinese patients receiving robotic mitral valve surgery. The procedure has been widely performed abroad, and it was first introduced in China 15 years ago.

This study confirmed that robotic MVRep is safe and effective, with 5 patients requiring sternotomy for postoperative bleeding. The clinical outcomes were satisfactory based on the short-term follow-up results. Follow-up TEE showed that the end-systolic diameter, end-diastolic diameter, and ejection fraction of the left ventricular improved postoperatively.

However, due to the current history of da Vinci surgery, further follow-up observations are needed to elucidate its long-term results (especially 5 years and beyond). In addition, due to the limited samples of this study, the data may be biased. With the development and improvement of da Vinci surgery, more patients will be included in future studies. Based on the current short- and mid-term efficacy, the da Vinci surgery should be further promoted in clinical practice so that more patients may be benefited.

None of the 120 patients in this study required intraoperative conversion to sternotomy for valve repair. However, patients should be aware that a sternotomy may be a second intraoperative option if the team faced intraoperative technical difficulties, switching the surgical approach could save lives. The goal of the strict training and certification of robotic mitral valve surgery teams of the hospital was to ensure patient safety and good clinical outcomes. Well-organized robotic mitral valve surgery as well as logical planning are essential to optimize patient safety. Annuloplasty ring implantation is the most time-consuming step in MVRep. The mastery of robotic surgical techniques significantly shortens the surgical duration. Our average surgical duration was 5.3 h.

The limitations of this study were that the patients were from the same center and the mean follow-up period was short. The robotic surgical system is only a surgical tool. The surgeries that the surgeon can perform depend on the surgeon's clinical experience rather than the robot. Therefore, the ultimate efficacy of robotic surgery should be further investigated in randomized trials.

Regarding establishing cardiopulmonary bypass for robotic mitral valve surgeries, the surgeon should be experienced in peripheral cannulation techniques using the femoral or axillary artery and femoral vein or jugular vein. Since robotic-assisted intracardiac surgery benefits from vacuum-assisted or dynamic venous drainage, cardiac surgery teams and perfusion teams should be well-experienced with such drainage techniques.

Increasingly challenging mitral valve procedures will be performed by robots in the future. Robotic surgery has been adopted in cases that were considered contraindications in the past, such as annular calcification and endocarditis, in recent years with good outcomes [18–20]. Future well-designed prospective randomized controlled trials should definitively assess the benefits of robotics in mitral valve surgery relative to open and other minimally invasive procedures to help clarify the advantages and disadvantages of robotic-assisted approaches.

## 5. Conclusions

The transesophageal echocardiography done as a follow-up to the surgery showed that the end-systolic diameter and the end-diastolic diameter of the left ventricular were all improved postoperatively. With the help of the da Vinci robot, MVRep is a safe and effective method of surgery for Chinese patients.

## Figures and Tables

**Table 1:** Characteristics of 120 patients receiving robotic MVRep.

Characteristics	N =120
Sex	
Male	78
Female	42
Age (years), mean ± SD	49.91 ± 12.12 (19-73)
Body mass index (kg/m^2^)	24.84 ± 5.59
Functional classification (NYHA)	2.52 ± 0.65
Comorbidities	
Hypertension	37
Atrial fibrillation	26
Diabetes mellitus	10
History of heart surgery (n, %)	3 (0.25%)
Preoperative LV end-systolic diameter (mm)	35.8 ± 6.2
Preoperative LV end-diastolic diameter (mm)	54.5 ± 7.5
Preoperative LV ejection fraction (%)	62.8 ± 6.6
Postoperative blood transfusion (n, %)	36 (30%)
Surgery duration (h)	5.34 ± 1.31
Cardiopulmonary bypass time (min)	152.32 ± 45.77
Aortic occlusion time (min)	95.13 ± 45.64
Bleeding volume (mL)	309.17 ± 87.87
Duration of ICU stay (h)	24.21 ± 11.85
Postoperative drainage volume (mL)	436.17 ± 65.77

Note: ICU, intensive care unit; LV, left ventricular; MVRep, mitral valve repair; NYHA, New York Heart Association; SD, standard deviation.

## Data Availability

The data used to support the findings of this study are available from the corresponding author upon request.
